# Bacterial Microbiome in the Phyllo-Endosphere of Highly Specialized Rock Spleenwort

**DOI:** 10.3389/fpls.2022.891155

**Published:** 2022-07-07

**Authors:** Valerie F. Masocha, Hongmei Liu, Pingshan Zhan, Kaikai Wang, Ao Zeng, Sike Shen, Harald Schneider

**Affiliations:** ^1^Centre for Integrative Conservation, Xishuangbanna Tropical Botanical Garden, Chinese Academy of Sciences, Beijing, China; ^2^University of Chinese Academy of Sciences, Beijing, China; ^3^School of Biological and Chemical Sciences, Pu’er University, Pu’er, China

**Keywords:** ferns, high-throughput sequencing, model organisms, phyllosphere, plant genomics, plant-growth promoting bacteria, symbiome phylogenetics, endophytic bacterial communities

## Abstract

Bacteria communities associated with plants have been given increasing consideration because they are arguably beneficial to their host plants. To understand the ecological and evolutionary impact of these mutualistic associations, it is important to explore the vast unknown territory of bacterial genomic diversity and their functional contributions associated with the major branches of the tree-of-life. Arguably, this aim can be achieved by profiling bacterial communities by applying high throughput sequencing approaches, besides establishing model plant organisms to test key predictions. This study utilized the Illumina Miseq reads of bacterial 16S rRNA sequences to determine the bacterial diversity associated with the endosphere of the leaves of the highly specialized rock spleenwort *Asplenium delavayi* (Aspleniaceae). By documenting the bacterial communities associated with ferns collected in natural occurrence and cultivation, this study discovered the most species-rich bacterial communities associated with terrestrial ferns reported until now. Despite the substantial variations of species diversity and composition among accessions, a set of 28 bacterial OTUs was found to be shared among all accessions. Functional analyses recovered evidence to support the predictions that changes in bacterial community compositions correspond to functional differentiation. Given the ease of cultivating this species, *Asplenium delavayi* is introduced here as a model organism to explore the ecological and evolutionary benefits created by mutualistic associations between bacteria and ferns.

## Introduction

Advancements in DNA sequencing technologies enable not only the study of the plant genomes (Marks et al., 2021) including the exploration of plant pan-genomes (Bayer et al., 2020), but also to unravel the cryptic contribution of arguably ubiquitous endophytic phytomicrobiomes—also called the plant endosphere or the holobiome of plants—to the adaption of land plants to their environment (Vorholt, 2012; Smith et al., 2017; Lyu et al., 2021; Cernava and Berg, 2022). By affecting traits of their host plants positively, some endophytic microorganisms, e.g., bacteria and fungi, support the functionality of ecological networks and the evolutionary success of land plants (Vandenkoornhuyse et al., 2015; Chen et al., 2020; Compant et al., 2020; Harrison and Griffin, 2020; Lyu et al., 2021; Tan et al., 2021). Furthermore, endophytic microbiomes have been increasingly considered to be treasure chests to discover bioactive metabolites (Gouda et al., 2016; Ek-Ramos et al., 2019; Wu et al., 2021; Cernava and Berg, 2022), to promote sustainable agriculture (Chen Q. L. et al., 2021; French et al., 2021), and to counteract the impact of the global climate change (Suryanarayanan and Shaanker, 2021; Perreault and Laforest-Lapointe, 2022). Unfortunately, the holobiome of the vast majority of plants is still awaiting to be discovered, which applies particularly to the bacterial diversity in the endo-phyllosphere—meaning the niche spaces inside the leaves of land plants (Vorholt, 2012; Khare et al., 2018; Cordovez et al., 2019; Harrison and Griffin, 2020; Chaudhry et al., 2021; Hawkes et al., 2021; Chialva et al., 2022). Many studies considered bacterial taxa enhancing plant defense (Matsumoto et al., 2021; Cernava and Berg, 2022; Shalev et al., 2022) besides other plant growth-promoting functions (Glick, 2012; Backer et al., 2018; Leontidou et al., 2020), but current studies expanded their focus on a wide range of ecological aspects such as the contribution of bacterial communities to the rapid adaption to local environments enforced by bacterial communities (Li et al., 2021) that coincide with diversification patterns (Abedlfattah et al., 2021) as argued in the SYMPHY proposal (Tripp et al., 2017) and the Plant Holobiont Theory (Lyu et al., 2021). Notable aspects studied include the enhanced resistance of plants to environmental stresses supported by endophytic bacteria, e.g., salt tolerance (Vaishnav et al., 2019) and heavy metal tolerance (Zhu et al., 2014; Xu et al., 2016; Gu et al., 2018; Banach et al., 2020; Yang et al., 2020; Antenozio et al., 2021) or even support the adaptation to extreme environments, e.g., deserts (Zhang et al., 2019) and karst habitats (Li F. et al., 2019). Endophytic bacteria have been also considered as promoters of horizontal gene transfer (Tiwari and Bae, 2020; Zilber-Rosenberg and Rosenberg, 2021). Finally, some endophytic bacteria associated with land plants may even play an essential role in mitigation of climate active gases such as isoprene and chloromethane to the atmosphere by forming communities degrading these gases within the phyllosphere (Crmbie et al., 2018; Jaeger et al., 2018; Kroeber et al., 2021).

Considering the rationale that mutualistic interactions between bacteria and plants provide unknown benefits, the study was designed to explore this untapped source by evaluating the prediction that the endo-phyllosphere of ferns hosts mostly unknown but highly diverse endophytic bacterial communities. These associations are expected to provide undiscovered benefits supporting the evolutionary success of this ancient plant lineage. This rationale considers recent findings supporting the hypothesis that bacteria associations of plants are constrained by genotypic and environmental factors (Morella et al., 2020), including plant neighborhood (Meyer et al., 2022) but also phylogenetic relationships (Harrison and Griffin, 2020; Lajoie and Kembel, 2021). An extensive review of published studies confirmed the lack of small number of surveys preventing careful analyses of ubiquity and trends in the microbial communities associated with the phyllosphere of ferns (see Supplementary Table 1). Arguably, the best-studied microbiomes of ferns are associated with the water fern *Azolla filiculloides* (Dijkhuzien et al., 2017; Banach et al., 2020), the arsenic hyperaccumulator *Pteris vittata* (Zhu et al., 2014; Xu et al., 2016; Gu et al., 2018; Yang et al., 2020; Antenozio et al., 2021), and the xeric fern *Pellaea calomelanos* (Mahlangu and Serepa-Dlamini, 2018a,b; Tshishonga and Serepa-Dlamini, 2019, 2020; Diale et al., 2021). The later studies explicitly focused on the genomes of cultivated endophytic bacteria, whereas other studies explored specifically antibacterial properties (Das et al., 2017a,b,c, 2018), growth-promoting abilities (Mukherjee et al., 2017; Rakshit and Sukul, 2021a,b), and ecological aspects in general (Jackson et al., 2006; de Araújo-Barros et al., 2010; Li F. et al., 2019). Enhancing our understanding of the endophytic bacterial communities are expected to specify the evolutionary history of the origin of the bacterial proteins such as Tma12 recovered in some fern genomes ferns arguably as a consequence of horizontal gene transfer (Li F. W. et al., 2019; Yadav et al., 2019). In summary, understanding the microbial communities associated with ferns will supply crucial information to understand the interactions between ferns and their environment, and significantly contributes to studies aiming to trace the origin of bacterial protein blueprints occurring in fern genomes.

**FIGURE 1:**
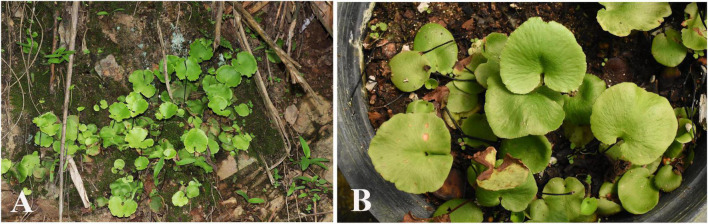
Sporophytes of the introduced model fern organism *Asplenium delavayi* **(A)** natural occurrences in Yunnan, **(B)** cultivated individuals at XTBG.

To tackle this pivotal gap in our understanding of fern biology, we intend to establish a fern model system to explore the ecological and evolutionary aspects of endophyllospheric bacterial communities. To achieve this, we employed a metabarcoding approach applying high throughput sequencing of 16S ribosomal RNA as now widely utilized in metagenomic studies of microbial communities (Langille et al., 2013; Xia et al., 2020) to identify the bacterial diversity associated with the leaf endosphere of the poorly known spleenwort *Asplenium delavayi* (Franch.) Copel. This morphologically distinct but rare species forms dense stands on mossy rock and ledges in canyons (Figure 1A). The distribution range includes southwestern China and adjacent regions in Bhutan, Northern India, Laos, Myanmar, and Thailand. The small-sized ferns produce several densely clustered leaves with a simple, orbicular to reniform shaped lamina that has a thick herbaceous texture (Figure 1A). Vegetative reproduction via proliferous roots facilitates not only the establishment of dense clusters in its natural habitats but also enable easy proliferation of individuals in culture conditions (Figure 1B). Relatively little is known about the ecology of this fern, but our own observations suggest a rapid turn-over of leaves in response to water stress during the dry season. Given the arguably stressful environment, a rich but highly variable community of endophytic bacteria is expected to occur in natural populations with the prominent bacterial representatives shared.

By generating a library of bacterial sequences of a variable region of the 16S rRNA gene, we scrutinized evidence to support or reject the following research predictions. (1) The leaves of this fern host a diverse community composed mainly of bacterial lineages previously reported to be associated with the holobiome of plants. (2) Despite distinct differences in the composition of the bacterial communities, a set of bacteria occurs in all accessions. (3) Differences in the microbiome coincides with functional differentiation. Finally, we aim to establish a protocol to explore the secrets of the leaf endosphere by using a cost-effective high throughput sequencing approach in combination with existing DNA databases and interpretation tools introduced to study microbial communities in general (Langille et al., 2013; Louca et al., 2016).

## Materials and Methods

### Sample Collection Protocol

The endosphere microbiota associated with the leaves of *Asplenium delavayi* were assessed using the high-throughput sequencing approach employing the MiSeq Illumina platform (Illumina, United States). Leaf samples were collected from two natural occurrences, accession AD0615 located in Yuanjiang County, Yuxi Prefecture, Yunnan, China (23°50′38.7″, 102°7′49.6″″, alt. 1,250 m) and accession AD0618 from Mojiang County, Pu’er Prefecture, Yunnan, China (23°33′12″, 101°33′8″, alt. 1,410 m). For comparison, we sampled leaves from plants cultivated at Xishuangbanna Tropical Botanical Garden, Chinese Academy of Sciences (accession number AD0620). No specific permissions were required for sampling in any location, nor were any endangered or protected species involved. Only a few leaves were sampled for each accession to avoid destroying the local populations, with one individual removed per location as a voucher deposited at the herbarium of XTBG (HITBC). For accessions AD0615 and AD0618, four subsamples were obtained by randomly collecting four individuals (1 to 4) per each forementioned locations. For each subsample, three leaves were aseptically cut by an ethanol sterilized scalpel, quickly put in sterile Ziploc plastic bags, and transferred on ice to our laboratory for further processing. For the cultivated accession AD0620, three leaves were sampled using the same procedure. To remove epiphylls, surface sterilization was carried out before microbial DNA extraction. Samples were sequentially washed with sterile Millipore water (30 s), followed by immersion in 70% (v/v) ethanol (2 min), 2.5% sodium hypochlorite solution (5 min) supplemented with 0.1% Tween 80, 70% (v/v) ethanol (30 s), and finally rinsing the samples five times with sterile Millipore water. We spread the last rinse water onto solidified LB media and incubated for 48 hours at 30°C to check if the sterilization was effective. If contamination was found, the surface sterilization procedure was repeated with some modifications of sterilant concentration. Finally, approximately 2.0 g of surface-sterilized leaves per sample were homogenized by portioning the samples into small fragments using a sterile scalpel and macerating them in sterile 10 mM phosphate saline (PBS) buffer (130 mM NaCl, 7 mM Na2HPO4, 3 mM NaH2PO4, pH 7.4).

### Library Preparation

Total genomic DNA was extracted from the surface-sterilized samples using the E.Z.N.A.™ Mag-Bind Soil DNA Kit (Omega, United States), following the manufacturer’s instructions. Each extraction’s DNA concentration and integrity were determined by using Qubit v.3.0 (Invitrogen, United States) and 1% agarose gel electrophoresis, respectively. To avoid amplification of chloroplast 16S sequences, we took advantage of the modified primer pair 799F/1492R specifically designed to mistarget the host plant organelle 16S RNA (Chelius and Triplett, 2001). PCR amplifications were carried out with the described mixtures—a final volume of 30 μL included 20 ng of gDNA mixed with 1μL of each primer at 10 μM and 12.5 μL of the 2 × Hieff^®^ Robust PCR Master Mix (Yeasen, Shanghai, China) and filled with 9 μL of PCR grade water—and the program as DNA denaturing at 94°C for 3 min, followed by 5 cycles of denaturing at 95°C for 30 s, annealing at 45°C for 30 s, elongation at 72°C for 30 s, then 20 cycles of denaturing at 95°C for 30 s, annealing at 55°C for 30 s, elongation at 72°C for 30 s and a final extension at 72°C for 5 min. PCR products were prepared for 16S sequencing following the Illumina Miseq system library preparation recommended protocol^1^. Second-round PCR was performed using the barcoded V5-V7 primers with Illumina adapters. The PCR products were purified using the AMPure XP magnetic beads and then pair-end sequenced using the MiSeq Illumina platform (Illumina, United States) at Sangon Biotech Co., Ltd. (Shanghai, China), according to the manufacturer’s instructions.

### Data Analyses

Raw Illumina reads were trimmed and filtered, applying a sliding window approach with a window size of 10 and a minimum quality of 20 as implemented in Trimmomatic v0.38 (Bolger et al., 2014). These reads were further processed by removing adapter sequences, and sequence pairs with one or both reads shorter than 200 bp using Cutadapt v 1.18 (Martin, 2011), followed by sequence assembling by employing Pear v0.9.8 (Zhang et al., 2014), and finally processed with Mothur v.1.43.0 (Schloss et al., 2009; Kozich et al., 2013). PCR and sequencing errors were reduced by removing ambiguous bases and homopolymers of more than eight nucleotides. The assembled sequences were aligned against the Silva SSU database release 138.1 (Quast et al., 2013) and de-noised by subjecting to a 2% pre-clustering allowing for up to 4 differences between sequences to reduce the contribution of possible sequencing errors. UCHIME (Edgar, 2013) was employed to remove potential chimeric sequences with > 3% errors before the remaining sequences were clustered into operational taxonomic units (OTUs) using a 97% identity threshold implemented in UC LUST (Wang et al., 2020). Raw sequencing data were deposited in NCBI’s Sequence Read Archive (SRA) in BioProject ID PRJNA794952. All recognized OTUs were classified into phylum, class, order, family, genus, and species levels using the Ribosomal Database Project Release 11.5 (Wang et al., 2007). We further analyzed the data with the packages phyloseq (McMurdie and Holmes, 2013) and vegan v. 2.5-7 (Oksanen et al., 2020) in R v3.6.0 (Core Development Team R, 2020). To examine the microbial communities associated with *A. delavayi* leaf samples, filtered reads were pooled into OTUs to identify the bacterial representatives. Rarefaction analyses using rarefy in vegan v. 2.5.-7 (Oksanen et al., 2020) were carried out to assess the richness and uniformity of the species composition. Phylogenetic relationships were estimated for all samples by conducting maximum likelihood analyses using RAxML v.8.2 (Stamatakis, 2014). The observed microbial diversity was explored by calculating five indices commonly used to assess microbial diversity by employing the mothur pipeline (Schloss et al., 2009; Kozich et al., 2013). The utilized indices were: ACE, Chao 1, Good’s Coverage, Shannon Diversity Index, and Simpson Diversity Index. The taxonomic distribution of microbial communities was visualized using the “ggplot2” v. 3.3.5 (Wickham et al., 2021), whereas Venn diagrams were generated to visualize the numbers of shared and unique OTUs among the three accessions. To contrast the species composition and functional differentiation among the nine libraries, the metagenomic functional content of the microbial communities was observed using the FAPROTAX 1.2.4 (Louca et al., 2016) and PICRUSt package (Langille et al., 2013) utilizing the KEGG database (Kanehisa et al., 2016, 2021) and COG databases (Tatusov et al., 2000). Principal coordinate analyses were carried out to detect differentiation between samples based on species composition or functional trait composition. The observed lineages of endophytic bacteria were taken into context of previously reported association of bacteria and ferns (Supplementary Table 1), including their plant association status as documented in PLaBAse v.1.01 (Patz et al., 2021).

## Results

**FIGURE 2:**
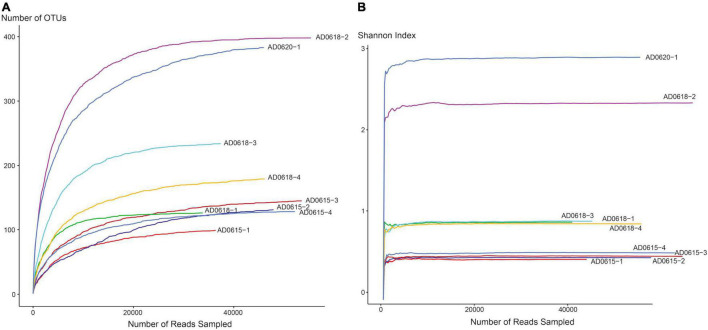
Rarefaction curves for OTU **(A)** and Shannon-Index **(B)** of leaf-associated bacterial communities in nine accessions of *Asplenium delavayi*. The *X*-axis shows the number of reads, whereas *Y*-axis shows the accumulation of the number of OTUs, and the increase of Shannon Index, respectively, for each of the nine accessions studied. Endpoints of graphs correspond to the number of reads per sample.

The total number of 16S bacterial sequences per sample ranged from 33,821 (AD0618-1) to 55,387 for AD0618-2 (Table 1), accumulating to a total number of sequences of 408,903. These sequences represented 831 OTUs, of which 28 were shared among the nine studied samples (Table 2). The proportion of these 28 shared OTUs varied between 51.4% (AD0620-1) to 99.2% (AD0615-1). Sequencing depth was sufficient to characterize the bacterial communities of all samples, given the graphs leveled off for both OTU rarefaction (Figure 2A) and Shannon-Index rarefaction (Figure 2B). The Good’s coverage of bacterial OTUs was consistently 1.0 for all samples studied (Table 1). Ignoring chloroplast sequences and unclassified sequences, the OTUs were assigned to 19 phyla, 34 classes, 74 orders, and 153 families (Table 3). A total of 30.63% of the OTUs and 3.72% of the sequences were not classified up to the family level (Table 4). Out of the 19 phyla, only four namely Actinobacteria, Bacteroidetes, Firmicutes, and Proteobacteria, were found across all accessions, and each included more than 10% of the OTUs recovered (Table 3 and Supplementary Tables 1, 2). These four phyla contributed 90% or more of the total number of sequences obtained (Tables 1, 3).

**TABLE 1:** Summary of the bacterial community observed in *Asplenium delavayi*.

AccNr	Location	SeqNum	OTUs	SHAN	ACE	CHAO	GOOD	SIMP	% FIRM	% PROT	% ACTI	% BACT	% OTH	% OTUs Shared	% OTUs Unique
AD0615-1	23°50′38″, 102°49′6″	36,402**	99	0.48	107.18	102.96	1	0.85	93.04	5.56	0.49	0.79	0.12	28.3*	8.1
AD0615-2	23°50′38″, 102°49′6″	47,947	131	0.50	162.10	155.23	1	0.84	92.85	6.01	0.34	0.5	0.3	21.4	6.1
AD0615-3	23°50′38″, 102°49′6″	53,555*	145	0.52	154.01	159.62	1	0.85	93.15	4.56	1.06	1.06	0.17	19.3	8.9
AD0615-4	23°50′38″, 102°49′6″	52,181	128	0.56	130.66	129.87	1	0.84	92.59	4.95	0.94	1.33	0.19	21.9	16.4
AD0618-1	23°33′12″, 101°33′8″	33,821**	126	0.92	127.24	129.75	1	0.72	86.23	9.21	1.92	2.45	0.19	22.2	12.7
AD0618-2	23°33′12″, 101°33′8″	55,387*	398*	2.35*	398.74*	398.46*	1	0.36**	67.08	11.79	5.63	5.55	9.95	7.0**	38.2*
AD0618-3	23°33′12″, 101°33′8″	37,403**	234	0.94	237.90	237.55	1	0.76	88.94	6.8	1.2	2.87	0.19	12	19.2
AD0618-4	23°33′12″, 101°33′8″	46,211	179	0.90	184.99	184.00	1	0.74	87.34	6.91	1.76	1.06	2.93	15.6	10.1
AD0620-1	Cultivated XTBG	45,996	383*	2.90*	396.22*	395.00*	1	0.21**	49.8	25.29	12.83	0.84	11.24	7.3**	45.4*

*The following information was recorded: Accession Number (AccN); Location given as geo-referenced data with the exception of AD0620-1 that was cultivated at XTBG; Number of Sequences obtained (SeqNum), Number of OTUs obtained (OTUs), Shannon Index (SHAN), Ace-Index (ACE), Chao-Index (CHAO), Good’s Coverage Index (GCI), Simpson’s Diversity Index (SIMP), Proportion of Sequences belonging to Firmicutes (%FIRM), Proportion of Sequences belonging to Proteobacteria (%PROT), Proportion of Sequences belonging to Actinobacteria (%ACTI), Proportion of Sequences belonging to Bacteriodetes (%BACT), Proportion of Sequences belonging to Others (% OTH), Proportion of the 28 OTUs shared by all samples (%OTUs shared), the proportion of OTUs unique to the sample (%OTUs unique).*

**TABLE 2:** Summary of the 28 OTUs occurring in all accessions.

						AD0615				AD0618				AD0620	
OTU	PHYLUM	CLASS	ORDER	FAMILY	GENUS	AD0615-1	AD0615-2	AD0615-3	AD0615-4	AD0618-1	AD0618-2	AD0618-3	AD0618-4	AD0620-1	Total
OTU84	Actinobacteria	Acidimicrobia	Acidimicrobiales	Lamiaceae	*Aquihabitans*	7	2	2	15	7	20	4	1	2	60
OTU42	Actinobacteria	Actinomycetia	Corynebacteriales	Gordoniaceae*	*Gordinia*	22	2	30	52	80	87	26	45	24	368
OTU58	Actinobacteria	Actinomycetia	Micrococcales	Dermacoccaceae	*Dermacoccus*	7	9	4	91	96	36	19	21	10	293
OTU24	Actinobacteria	Actinomycetia	Micrococcales	Infrasporangiaceae*	*Tetrapshaera*	17	7	59	26	65	49	25	35	17	300
OTU51	Actinobacteria	Actinomycetia	Micrococcales	Micobacteriaceae*	*Microbacterium*	10	9	29	24	58	50	31	17	29	257
OTU27	Actinobacteria	Actinomycetia	Micrococcales	Micrococcaceae*	*Kocuria*	25	13	47	84	99	112	54	50	29	513
OTU26	Actinobacteria	Actinomycetia	Propiniobacteriales	Propiniobacteriaceae*	*Propiniobacterium*	10	12	31	45	10	271	100	8	23	510
OTU13	Bacteroidetes	Flavobacteria	Flavobacteriales	Weeksellaceae*	*Chryseobacterium*	109	24	139	108	153	219	137	81	27	997
OTU6	Bacteroidetes	Flavobacteria	Flavobacteriales	Weeksellaceae*	*Epilithonimonas*	152	90	218	403	562	317	178	178	65	2163
OTU38	Bacteroidetes	Flavobacteria	Flavobacteriales	Weeksellaceae*	*Chryseobacterium*	6	7	32	30	30	36	11	22	11	185
OTU1	Firmicutes	Bacilii	Bacillales	Bacilliaceae*	*Bacillus*	33,477	44,023	49,407	47,872	28,679	32,634	32,562	39,643	19,891	3,28,188
OTU5	Firmicutes	Bacilii	Bacillales	Bacilliaceae*	*Terribacillus*	329	292	384	311	284	220	275	345	102	2542
OTU80	Firmicutes	Bacilii	Lactobacillales	Streptococcaceae	*Streptococcus*	25	3	34	25	29	40	23	81	7	267
OTU75	Firmicutes	Bacilii	Lactobacillales	Streptococcaceae	*Streptococcus*	7	13	3	2	1	8	18	33	14	99
OTU45	Proteobacteria	Alphaprotobacteria	Caulobacterales	Caulobacteraceae*	*Brevundimonas*	21	4	35	47	58	62	29	18	181	455
OTU11	Proteobacteria	Alphaprotobacteria	Rhizobiales	Bradyrhizobiaceae*	*Unclassified*	252	362	277	368	539	410	286	277	784	3555
OTU9	Proteobacteria	Alphaprotobacteria	Rhizobiales	Bradyrhizobiaceae*	*Unclassified*	122	181	97	187	253	200	175	149	62	1426
OTU90	Proteobacteria	Alphaprotobacteria	Rhodobacterales	Rhodobacteraceae	*Paracoccus*	12	1	15	28	4	14	17	9	4	104
OTU17	Proteobacteria	Alphaprotobacteria	Sphingomonadales	Sphingomonadaceae*	*Sphigomonaes*	10	61	8	26	9	27	16	177	1506	1840
OTU138	Proteobacteria	Alphaprotobacteria	Sphingomonadales	Unclassifiedied	*Unclassified*	1	4	3	13	7	7	6	5	2	48
OTU3	Proteobacteria	Betaprotobacteria	Burkholderiales	Burckholderiaceae*	*Burckholderia*	1232	1593	1343	993	1196	943	682	872	334	9188
OTU10	Proteobacteria	Betaprotobacteria	Burkholderiales	Burckholderiaceae*	*Ralstonia*	144	147	209	231	230	440	90	79	122	1692
OTU16	Proteobacteria	Betaprotobacteria	Burkholderiales	Comanoadacaee*	*Pelomanes*	35	38	74	92	173	119	49	42	143	765
OTU18	Proteobacteria	Gammaprotobacteria	Enterobacteriales	Erwiniaceae*	*Pantoea*	50	62	42	228	136	270	35	88	81	992
OTU176	Proteobacteria	Gammaprotobacteria	Enterobacteriales	Erwiniaceae*	*Unclassified*	14	2	2	12	15	75	34	10	5	169
OTU57	Proteobacteria	Gammaprotobacteria	Moraxalleles	Moraxellaceae*	*Unclassified*	11	1	9	4	1	43	32	15	128	244
OTU65	Proteobacteria	Gammaprotobacteria	Pseudomonadales	Pseudomonadaceae*	*Pseudomonas*	2	1	20	25	29	4	16	8	17	122
OTU40	Proteobacteria	Gammaprotobacteria	Unclassified	Unclassified	*Unclassified*	11	28	44	17	7	28	25	21	11	192
			**Total Sequences contributed by shared OTUs**			36,120	46,991	52,597	51,359	32,810	36,741	34,955	42,330	23,631	3,57,534
			**Total Sequences obtained**			36,402	47,947	53,555	52,181	33,821	55,387	37,403	46,211	45,996	4,08,903
			**Proportion contributed by shared OTUs**			99.2	98	98.2	98.4	97.1	66.3	93.5	91.6	51.4	87.4

The following information was recorded: Number of OTU (OTU), the next five columns provided the classification according to the taxonomy browser of the Genbank; the following 14 columns showed the summary of the number of sequences obtained for each OTU in each accession. The final column (total) summarized the number of sequences contributed by each OTU. The three last rows summarized the total number of sequences contributed by the shared OTUs for each accession, all sequences obtained for each accession, and finally, the proportion (%) contributed by the shared OTUs. *Families recorded to comprise taxa involved in Plant Growth Promotion Activity.

**TABLE 3:** Summary of the phyla diversity observed in *Asplenium delavayi*.

Bacteria Phyla	Shared OTUs %	Near share OTUs %	Total OTUs %	NrSeq %	NrAcc %	Classes	Orders	Families
Acidobacteria	0	0	1.09	0.013	55.6	1uc	NA	NA
Actinobacteria	24.997	28.572	18.035	2.99	100	6C + uc	19O + 1uo	34F + 5uf
Armatimonadetes	0	0	0.605	0.064	33.3	1uc	NA	NA
Bacteroidetes	10.714	14.286	16.827	1.87	100	7C + 1uc	7O + 1uo	19F + 4uf
Bacteria candidate Phyla	0	0	1.453	0.036	88.9	1C	1O	1F
Chlamydiae	0	0	0.605	0.009	44.4	1C + 1uc	1O	1F
Chloroflexi	0	0	0.151	0.001	44.4	3C	3O	3F
Deinococcus-Thermus	0	4.762	0.847	0.042	77.8	1C	2O	2F
Fibrobacteres	0	0	0.121	0.001	11.1	1C	1O	1F
Firmicutes	14.286	9.524	12.829	83.39	100	4C + 1uc	6O + 3uo	23F + 2uf
Fusobacteria	0	0	0.121	0.007	55.6	1C	1O	1F
Ignavibacteriae	0	0	0.847	0.015	88.9	1C	1O	1F
Nitrospirae	0	0	0.121	0.001	11.1	1C	1O	1F
Planctomycetes	0	0	0.847	0.009	44.4	1C + 1uc	2O + 1uo	2F
Proteobacteria	49.997	42.858	37.644	11.33	100	6C + 1uc	28O + 5uo	60F + 6uf
Spirochaetes	0	0	0.242	0.006	22.2	1C	1O	1F
Synergistetes	0	0	0.242	0.003	11.1	1C	1O	1F
Tenericutes	0	0	0.121	0.017	22.2	1C	1O	1F
Verrucomicrobia	0	0	0.484	0.009	44.4	1C + uc	1O	1F
unclassified	0	0	6.053	0.968	100	NA	NA	NA
unclassified	0	0	6.053	0.968	100			

*The following information was recorded: Bacteria Phyla according to the Taxonomy Browser of Genbank; proportion of the 28 shared OTUs (Shared OTUs), proportion of OTUs shared by at least 7 but not all 9 accessions (nearly shared OTUs), proportion of the total number OTUs (total OTUs), proportion of number of sequences (NrSeq), proportion of the number of accessions with the phylum recorded (NrAcc). The final three columns summarized the classification of the OTUs into classes, orders, and families by showing the number of classes (C), orders (O), and families (F), plus the number of unclassified classes (uc), order (uo), and families (uf). NA = not applicable.*

**TABLE 4:** Summary of OTUs and Sequences that were not classified to the family rank.

Unclassified	OTU %	Sequences %
Bacteria	6.05	0.97
Phyla	7.35	0.31
Class	7.84	0.26
Order	9.39	2.18
Total	30.63	3.72

*Proportions were given as follows: Bacteria = blast results showed only the association as a Bacteria 16S sequence; Phylum = blast results provided only assigned to phylum; Class = blast results provided only assigned to class; Order = blast results provided only assigned to order. The final row (Total) summarized the proportion of unclassified OTUs and Sequences that were not classified at least to the family rank.*

The four subsamples of accession AD0615 showed highly similar bacterial diversity as documented by the average values and standard errors of four biodiversity indices, namely Shannon-Index = 0.52 ± 0.02, ACE-Index = 138.49 ± 12.38, Chao-Index 136.92 ± 0 = 13.08, and Simpson-Diversity Index = 0.85 ± 0.003. These samples show a notable dominance by Firmicutes with an average contribution of 92.91% ± 0.12 (Table 1). In contrast, subsamples of AD0618 showed increased variation in the bacterial diversity as documented by average values of the four biodiversity indices, namely Shannon-Index = 1.28 ± 0.36, ACE-Index = 2377.44 ± 58.39, Chao-Index 2,377.44 ± 58.01, and Simpson-Diversity0Index = 0.65 ± 0.10. These values coincided with a highly reduced contribution of Firmicutes with an average contribution of 82.40% ± 5.13. The cultivated accession AD0620 strongly diverged from the two wild collections (Table 1), especially by the strong decline of Firmicutes with 49.80% (Table 1).

**FIGURE 3:**
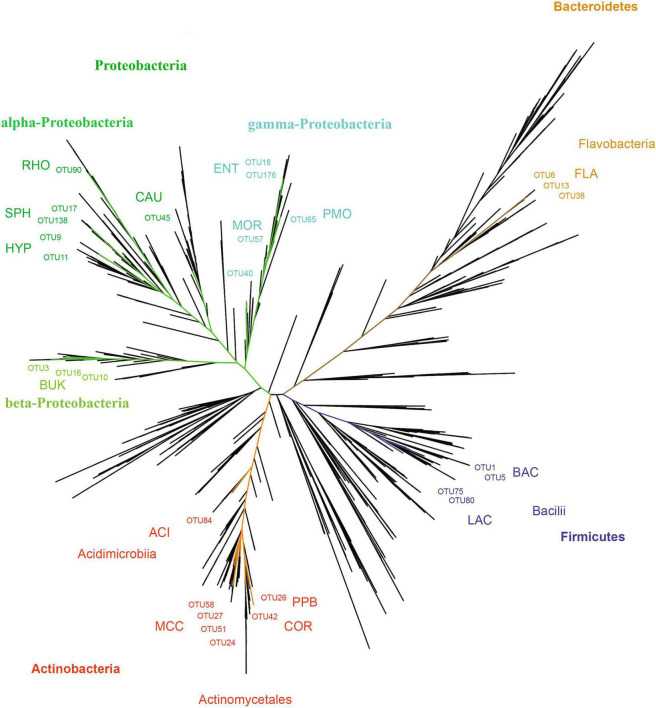
Unrooted phylogram showing the relationships of OTUs. The 28 OTUs shared among all samples are marked and classified. The main lineages (phylum) detached are marked using a color code: Actinobacteria (red), Bacteroidetes (brown), Firmicutes (blue), Protobacteria (brown). Besides phyla, the placenta of classes and orders are marked with the later abbreviated: ACI = Acidimicrobiales, BAC = Bacillales, BUK = Burkholderiales, CAU = Caulobacterales, COR = Corynebacteriales, ENT = Enterobacteriales, FLA = Flavobacteriales, HYP = Hyphomicrobiales, LAC = Lactobacillales, MCC = Micrococcales, MOR = Moraxellales, PMO = Pseudomonadales, PRO = Propionibacteriales. RHO = Rhodobacterales, SPH = Sphingomonadales.

**FIGURE 4:**
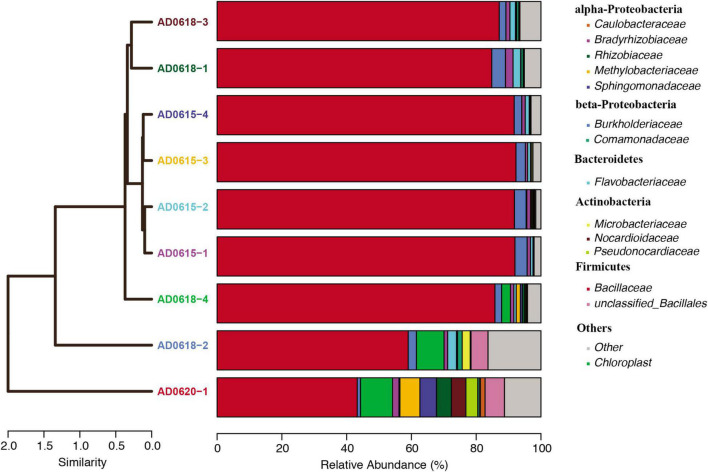
Relative abundance of bacterial lineages plotted for each accession. The cluster analyses illustrated the similarity among lineages visualized as a tree, whereas the relative abundance blocks visualized the proportion of each major bacterial lineage recovered. Color code showed on the right side.

**FIGURE 5:**
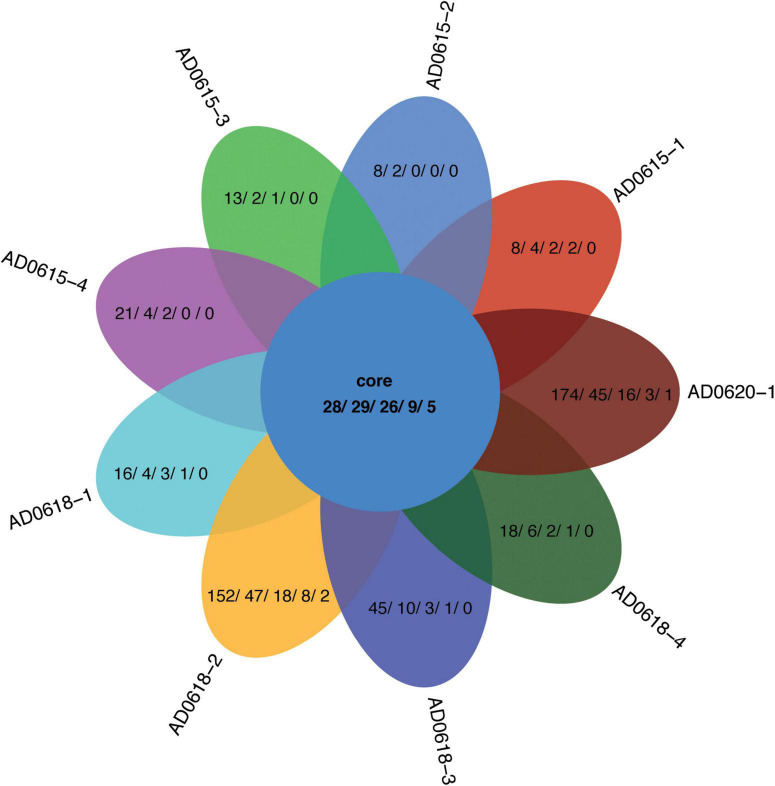
Venn diagram showing the distribution contrast of the OTUs shared among all accessions and the unique OTUs per accession not shared with all other accessions. Numbers given represent the OTU/Phylum/Class/Order/Family, respectively.

In total, 28 OTUs were shared among all samples obtained, although their frequency varied substantially (Table 1). These 28 OTUs represented four phyla, seven classes, 15 orders, 20 families, and 21 genera (Figure 3 and Supplementary Table 2). Accessions with a Shannon Index > 1.00, namely AD0618-2 and AD0620, also had the lowest frequency of the shared OTUs. The frequency of unique OTUs was negatively correlated with the 28 shared OTUs with r^2^ = 0.701 and p = 0.004 (Table 1 and Figures 4, 5). These trends altered the composition of the phyla, classes, order, and family, respectively (Figure 4). The class formed by unclassified sequences was most predominant in AD0618-2 and AD0620-1. The number of shared OTUs suggests conservation of the endophytic community in this taxon, and only two accessions showed evidence for substantial expansion of bacterial diversity.

**FIGURE 6:**
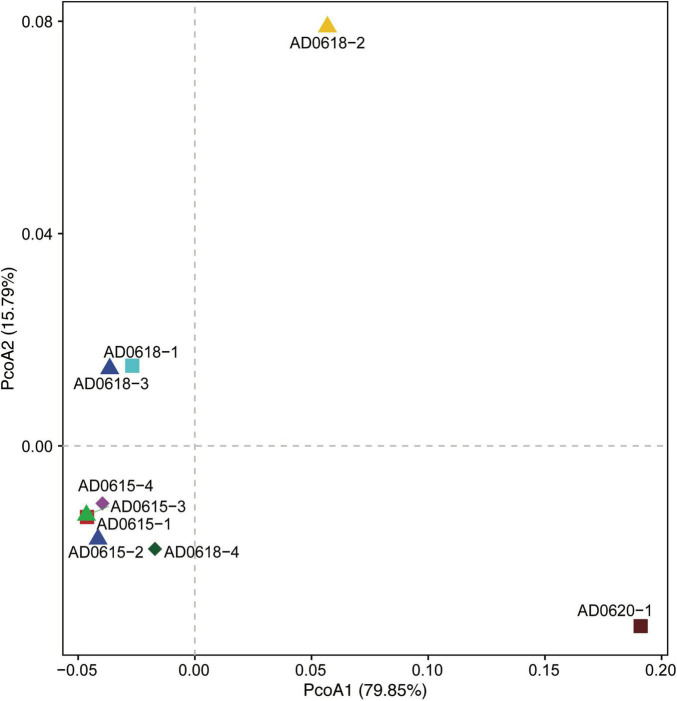
Principal Coordinate Analyses illustrating the similarities and differences of the bacterial communities’ diversity among the nine accessions studied. Axis1 explains 79.85%, and Axis2 explains 15.79% of the total variation. All accessions of ADO615 show high similarity, whereas AD0618 show a stronger differentiation among its four accessions, mainly along PCoA2.

**FIGURE 7:**
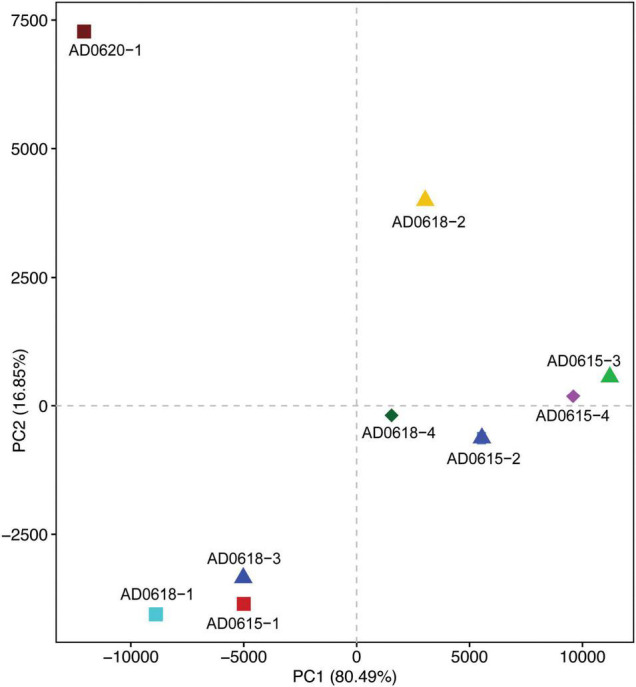
Principal Component Analyses illustrating the similarities and distinction of the functional attributes of the bacterial communities among the nine accessions studied. The shown scatter plot was based on FAPROTAX. Axis1 explained 80.49% and Axis2 explained 16.85% of the variation. Chemoheterotrophy was the main contributor to axis1 besides methanol/methyl and nitrogen processing pathways, which were also main contributors to axis 2 besides fermentation. Scatter plots based on PICRUSt cog and ko analyses showed highly similar results (results not shown).

Contrasting functional differentiation with diversity richness patterns recovered functional differentiation among the individuals collected from the same location. In both analyses, AD0620-1 and AD0618-2 were highly isolated, consistent with the strongly differentiated community composition (Figures 6, 7). In diversity analysis, AD0618-1 and AD0618-3 were highly similar and separated from AD0618-4 that clustered together with the four subsamples of accession AD0615 (Figure 5). Functional analyses utilizing FAPROTAX or PICRUST recovered AD0618-4 separated from other samples of AD0618 by showing similar functional trends as AD0615-1 and AD0615-2, AD0615-3, and AD0615-4. However, in contrast to diversity patterns, AD0615-1 was highly similar to AD0618-3 in the functional analyses (Figure 6). Functional analysis via FAPROTAX recovered a large portion of undefined functional hits (Table 5) but predicted functions shared among accessions belonging to pathways involving chemoheterotrophy, fermentation, methylotrophy and nitrogen household (Table 5). Consistent with the expanded diversity, AD0618-2 and AD0620 showed distinct trends such as reduction of the proportion of the functional group “other” and the highest proportion of hits for “Chemoheterotrophy” (Table 5), besides hits to functional groups such as “cellulolysis”, “manganese oxidation”, and “thiosulfate respiration” only found in these accessions (Table 5).

**TABLE 5:** Summary of the FAPROTAX-based inference of functional differentiation.

FAPROTAX functional group	AD0615-1	AD0615-2	AD0615-3	AD0615-4	AD0618-1	AD0618-2	AD0618-3	AD0618-4	AD0620-1	TotHit	TotHit%	NrAccPr	UniLoc	CFFG
Other*	34549	45643	51266	49640	30712	44107	34591	41722	30359	362589	79.62	9	–	
Chemoheterotrophy*	1684	2109	2061	2114	2475	5657	2417	3010	10567	32094	7.05	9	–	
Aerobic Chemoheterotrophy*	1560	1828	1893	1702	2037	2825	1385	1701	7072	22003	4.83	9	–	
Chloroplasts*	0	94	0	2	0	4728	1	1258	4549	10632	2.33	6	–	
Fermentation*	122	113	168	404	428	2794	1010	660	162	5861	1.29	9	–	
Methylotrophy	19	170	20	39	20	65	54	661	3347	4395	0.97	9	–	MET
Methanol Oxidation	19	170	20	36	20	39	47	627	3101	4079	0.90	9	–	MET
Ureolysis	18	179	0	10	67	22	12	595	2993	3896	0.86	8	–	NIT
Nitrate Reduction	177	93	249	459	604	525	261	206	97	2671	0.59	9	–	NIT
Aromatic Compound Degradation	24	14	55	71	137	175	79	68	1119	1742	0.38	9	–	
Respiration of Sulfur Compounds **	0	0	0	0	8	398	153	4	0	563	0.12	4	AD0618	SUL
Sulfate Respiration **	0	0	0	0	8	350	121	4	0	483	0.11	4	AD0618	SUL
Animal Parasites or Symbionts	8	11	30	48	48	153	90	11	17	416	0.09	9	–	
Manganese Oxidation **	0	0	0	0	0	0	0	0	344	344	0.07	1	AN02620	
Hydrocarbon Degradation	0	0	0	3	10	26	13	34	246	332	0.07	6	–	HYD
Human Pathogens all	6	11	30	29	48	109	61	9	17	320	0.07	9	–	
Methanotrophy	0	0	0	3	0	26	7	34	246	316	0.07	5	–	MET
Nitrate Respiration	17	2	20	30	20	58	45	17	7	216	0.05	9	–	NIT
Nitrogen Respiration	17	2	20	30	20	58	45	17	7	216	0.05	9	–	NIT
Nitrogen Fixation	13	6	0	2	64	25	7	9	89	215	0.05	8	–	NIT
Nitrate Denitrification	17	2	20	30	20	38	45	17	7	196	0.04	9	–	NIT
Nitrite Denitrification	17	2	20	30	20	38	45	17	7	196	0.04	9	–	NIT
Nitrous Oxide Denitrification	17	2	20	30	20	38	45	17	7	196	0.04	9	–	NIT
Denitrification	17	2	20	30	20	38	45	17	7	196	0.04	9	–	NIT
Nitrite Respiration	17	2	20	30	20	38	45	17	7	196	0.04	9	–	NIT
Iron Respiration	0	1	0	0	0	132	40	14	3	190	0.04	5	–	
Dark Hydrogen Oxidation	17	2	20	30	20	22	32	12	7	162	0.04	9	–	
Dark Oxidation of Sulfur Compounds	0	5	0	0	0	0	1	52	53	111	0.02	4	–	SUL
Human Gut	3	0	6	9	8	38	20	5	4	93	0.02	8	–	
Mammal Gut	3	0	6	9	8	38	20	5	4	93	0.02	8	–	
Sulfur Respiration	0	0	0	0	0	42	32	0	0	74	0.02	2	AD0618	SUL
Predatory or Exoparasitic	0	0	10	0	0	6	0	8	48	72	0.02	2	AD0618	
Sulfite Respiration	0	0	0	0	0	46	20	0	0	66	0.01	2	AD0618	SUL
Intracellular Parasites	0	0	0	0	0	16	9	7	4	36	0.01	3	AD0618	
Photoheterotrophy	2	0	0	1	0	13	8	8	3	35	0.01	5	AD0618	
Phototrophy	2	0	0	1	0	13	8	8	3	35	0.01	6	AD0618	
Aliphatic Non-Methane Hydrocarbon Degradation	0	0	0	0	10	0	6	0	0	16	0.00	2	AD0618	HYD
Aromatic Hydrocarbon Degradation	0	0	0	0	10	0	6	0	0	16	0.00	2	AD0618	HYD
Thiosulfate Respiration	0	0	0	0	0	6	0	0	0	6	0.00	1	AD0618	SUL
Aerobic Nitrite Oxidation	0	0	0	0	0	0	0	0	5	5	0.00	1	AN02620	NIT
Nitrification	0	0	0	0	0	0	0	0	5	5	0.00	1	AN02620	NIT
Cellulolysis	0	0	0	0	0	3	0	0	0	3	0.00	1	AD0618	
AccNrHits	38345	50463	55974	54822	36882	62705	40826	50851	64513	455381				
AccConHit%	8.4	11.1	12.3	12	8.1	13.8	9	11.2	14.2					

42 FARPROTAX Functional Groups (FFG) were detected with at least one hit. Column 1 list these 42 FFG according to the number of total hits (TotHit). The number of hits were given for each accession by each functional group. The accumulated number of hits were given in the row AccNrHits, whereas the row AccConHit% summed the contribution of hits contributed by each accession to the total number of hits in percent. The column TotHit reported the sum of all hits across all accessions, whereas the column TotHit% summarized the proportion of hits of each FFG contributed to the total number of hits in percent. Column NrAccPr reported the number of accessions with hits per FFG, whereas Column UniLol marked FFGs with hits only recorded at one location. In this case the location was named. *Marked FFGs contributing more than 1% of the total hits, whereas ** marked FFGs contributing > 0.05% of the total hits but were restricted to accessions from one location. To enable further characterization of the functional aspects of the endophytic bacterial communities, the 42 FFGs were classified into functional classes (CFFG) of interest: HYD = putative association with hydrocarbon degradation, MET = putative association with Methylation, NIT = putative association with Nitrogen cycle, SUL = putative association with Sulfur cycle.

## Discussion

The leaves of the spleenwort *Asplenium delavayi* host a rich endophytic bacterial community exceeding any previous record of fern bacterial association considering the number of phyla, classes, orders, families-, and genera documented (see Supplementary Table 1). This result may be partly explained by approaches employed and the research foci selected in previous studies. None of the previous studies explicitly targeted the documentation of the bacteria communities colonizing the leaf endosphere using a high throughput sequencing approach. Instead, several studies employed the more traditional approach to cultivate bacteria to discover strains with antimicrobial activities (Das et al., 2017a,b,c, 2018), or to document the variation used by growth regimes (de Araújo-Barros et al., 2010). 16S rRNA sequences were utilized in several studies, but these studies were designed to address particular research foci such as the discovery of chloromethane degraders (Jaeger et al., 2018; Kroeber et al., 2021) or heavy-metal resistant strains (Zhu et al., 2014; Xu et al., 2016; Gu et al., 2018; Antenozio et al., 2021). Two studies are arguably most similar to our study in their research focus and strategy. These studies aimed to document the bacterial communities associated with the leaves of resurrection fern *Pleopeltis polypodioides* (Jackson et al., 2006) and the xeric fern *Pellaea calomelanos* (Mahlangu and Serepa-Dlamini, 2018a), respectively. However, only the study of *Pleopeltis polypodioides* (Jackson et al., 2006) recovered also a notable diverse bacterial community, including 7 phyla, 11 classes, 15 orders, and 20 families (Supplementary Table 1). Similar to our study, 16S rRNA sequences were targeted, but the sequencing protocols available in 2016 were arguably less potent than the approaches available in 2021. Furthermore, we have to point out the minimal number of fern species studied, namely 17 species (Supplementary Table 1) and the lack of evidence for several orders of ferns. Nevertheless, discovering a rich endophytic bacterial community associated with *Asplenium delavayi* provides a unique opportunity to explore the ecological and evolutionary aspects of these associations by establishing this easily cultivated fern species as a model system.

In this context, it is crucial to note that our study recovered a set of OTUs found in all samples studied. These 28 OTUs not only dominated the communities of all samples except for AD0618-2 and AD0620-2, but also contributed about half of the sequences in those accessions (Table 2). The vast majority of these OTUs belong to families repeatedly reported to be associated with plants, including the phyllosphere, and are considered to comprise “plant growth-promoting bacteria” (Glick, 2012; Backer et al., 2018; Patz et al., 2021). In recent years, increasing consideration has been given to the utilization of bacteria (Sessitsch et al., 2019), for example to improve crop production on nutrient-poor soils instead of using artificial fertilizers (Chen L. et al., 2021). To improve our capability to use these interactions, studies are not only needed to focus on economically important plants, but assessments of the diversity and evolutionary significance of these associations are also essential. Major challenges observed in this study are the large amount of sequence data lacking a classification due to the limited exploration of the 16S rRNA diversity of bacteria as well as the limitations of functional trait databases such as FAPROTAX (Louca et al., 2016). Nevertheless, the discovered bacterial communities associated with *Asplenium delavayi* showed potentially involvement in nitrogen household and methane degrading (Table 5). Both issues were highlighted as major topics in studies on “plant growth-promoting bacteria” (Li et al., 2021), management of environments in a globally warming world (Jaeger et al., 2018; Kroeber et al., 2021), and set up of new strategies for disease prevention (Cernava and Berg, 2022). However, not all the bacteria associated with fern leaves may provide benefits and some may even represent pathogens (Kloepper et al., 2013). To unravel the complexities and dynamics of these mutualistic interactions, we herein propose a need to carry out carefully designed experiments targeting *Asplenium delavayi* as a model system. Despite the limited numbers of samples, this study showed variation in richness and composition likely associated with differences in the environment, e.g., natural habitat versus cultivated conditions.

Besides the commonly associated lineages such as Actinobacteria, Bacteroidetes, Firmicutes, and Proteobacteria, some accessions comprised a small portion of sequences identified as belonging to the phylum Chloroflexi. This poorly known lineage of bacteria has been proposed to be the origin of the Tma12 insecticidal protein found in ferns (Li F. W. et al., 2019). The discovery of Chloroflexi occurring in the endophyllosphere of ferns provides additional support to the hypothesis of a horizontal gene transfer of this gene from bacteria to ferns. In general, endophytic bacteria have been considered to support HGT events in plants (Tiwari and Bae, 2020; Zilber-Rosenberg and Rosenberg, 2021), but relatively few reports can be directly associated to support this hypothesis. Thus, we suggest giving enhanced attention to the occurrence of bacterial sequences in sequencing reads obtained in the whole genome sequencing project. Instead of deleting as a result of contaminations, they may be best considered highly informative evidence to study the bacterial communities associated with ferns.

By reviewing the published evidence of endophytic bacterial communities associated with ferns, we clearly showed an insufficient grasp on the potential of the crucial ecological and evolutionary contribution of these associations. Together with our new records, assessments are available for only 17 species belonging to 15 genera, 11 families, and six orders. Furthermore, previous studies have rarely aimed to identify all the bacteria associated with ferns. In turn, our study showed the potential of a high throughput sequencing approaches enabled by the advancements of genome sequencing to document the bacterial diversity associated with ferns including the capacity to carry out well designed experiments to test variation of bacterial communities associated with fern leaves under different environmental conditions.

## Conclusion

*Asplenium delavayi* is an outstanding candidate to be utilized as a model system to carry out studies with the aim to improve our understanding of the ecological and evolutionary significance of endophyllospheric bacterial associations of ferns and plants in general. For the first time, a highly diverse bacterial community was recorded for a terrestrial fern, whereas previous studies exploring fern-bacterial communities focused mainly on the water fern *Azolla* (Dijkhuzien et al., 2017; Li F. W. et al., 2019; Banach et al., 2020). The latter has been considered a highly specialized and unique example for symbiotic interactions between ferns and bacteria, including coinciding diversification of bacteria and these remarkable water ferns (Tripp et al., 2017). Still, our new findings suggest a more widespread and potential ubiquitous occurrence of mutualistic interactions between ferns and bacteria as considered in the concepts of the plant holobiont theory (Lyu et al., 2021), and symbiome phylogenetics (Tripp et al., 2017). Furthermore, the study supported the expectation that high throughput sequencing approaches enable the rapid assessment of bacterial communities associated with plants, including differentiation among accessions collected from different localities and cultivation conditions. Also, in as much as the work made a substantial descriptive profiling of the present organisms and their prospective functions, it did not give a comprehensive insight of whether these organisms are active members of the microbiome. Therefore, we suggest the extension of our current findings by integrating additional meta-transcriptomics with microbiome data as to have a deeper understanding of the microbes’ functions.

## Data Availability Statement

The datasets presented in this study can be found in online repositories. The names of the repository/repositories and accession number(s) can be found below: https://www.ncbi.nlm.nih.gov/, PRJNA794952.

## Author Contributions

HL and HS designed the project. VM designed and carried out the DNA library preparation and analyses. PZ, KW, AZ, and SS collected the populations occurring in Yunnan. HS and HL wrote the manuscript. All authors approved the final manuscript before submission and listed have made a substantial, direct, and intellectual contribution to the study.

## Conflict of Interest

The authors declare that the research was conducted in the absence of any commercial or financial relationships that could be construed as a potential conflict of interest.

## Publisher’s Note

All claims expressed in this article are solely those of the authors and do not necessarily represent those of their affiliated organizations, or those of the publisher, the editors and the reviewers. Any product that may be evaluated in this article, or claim that may be made by its manufacturer, is not guaranteed or endorsed by the publisher.

## Footnotes

1
https://support.illumina.com
